# Comparative Assessment of Heavy Metals in Drinking Water Sources in Two Small-Scale Mining Communities in Northern Ghana

**DOI:** 10.3390/ijerph120910620

**Published:** 2015-08-28

**Authors:** Samuel J. Cobbina, Abudu B. Duwiejuah, Reginald Quansah, Samuel Obiri, Noel Bakobie

**Affiliations:** 1 Department of Ecotourism and Environmental Management, Faculty of Renewable Natural Resources, University for Development Studies, P.O. Box TL 1882, Nyankpala 233, Ghana; E-Mails: abalu096@gmail.com (A.B.D.); nbakobie@gmail.com (N.B.); 2 School of the Environment and Safety Engineering, Jiangsu University, Xuefu Rd. 301, Zhenjiang 212013, China; 3 Biological, Environmental and Occupational Health Sciences, School of Public Health, College of Health Sciences, University of Ghana, P.O. Box LG 25 Legon, Accra 233, Ghana; E-Mail: yaw121@yahoo.co.uk; 4 Department of Immunology, Noguchi Memorial Institute for Medical Research, College of Health School of Public Health, University of Ghana, Legon 233, Ghana; 5 Environmental Chemistry Division, CSIR-Water Research Institute, P.O. Box A38, Accra 233, Ghana; E-Mail: obirisamuel@gmail.com

**Keywords:** arsenic, mercury, Northern Ghana, small-scale mining, water quality

## Abstract

The study assessed levels of heavy metals in drinking water sources in two small-scale mining communities (Nangodi and Tinga) in northern Ghana. Seventy-two (72) water samples were collected from boreholes, hand dug wells, dug-out, and a stream in the two mining communities. The levels of mercury (Hg), arsenic (As), lead (Pb), zinc (Zn), and cadmium (Cd) were determined using an atomic absorption spectrophotometer (AAS). Mean levels (mg/l) of heavy metals in water samples from Nangodi and Tinga communities were 0.038 and 0.064 (Hg), 0.031 and 0.002 (As), 0.250 and 0.031 (Pb), 0.034 and 0.002 (Zn), and 0.534 and 0.023 (Cd), respectively, for each community. Generally, levels of Hg, As, Pb, Zn, and Cd in water from Nangodi exceeded the World Health Organisation (WHO) stipulated limits of 0.010 for Hg, As, and Pb, 3.0 for Zn and 0.003 for Cd for drinking water, and levels of Hg, Pb, and Cd recorded in Tinga, exceeded the stipulated WHO limits. Ingestion of water, containing elevated levels of Hg, As, and Cd by residents in these mining communities may pose significant health risks. Continuous monitoring of the quality of drinking water sources in these two communities is recommended.

## 1. Introduction

Water supply systems and drinking water inaccessibility in developing countries is a global concern that calls for immediate action. About 884 million people in the world still do not get their drinking water from approved sources, and almost all of these people are in developing regions [1]. Providing quality drinking water to all citizens who are deprived of access to water will serve as the breaking point of poverty alleviation in most developing countries, especially in Africa, where substantial amounts of national budgets are used to treat preventable waterborne diseases [2]. Having reliable drinking water is now recognised by United Nations as a human right.

A heavy metal is any metallic element that has a relatively high density and is toxic or poisonous even at low concentrations [3]. Heavy metals exist as natural constituents of the earth crust and are persistent environmental contaminants, because they cannot be degraded or destroyed [3,4]. Whilst these elements occur naturally they are often bound up in inert compounds. However, their concentrations have increased several-fold as a result of anthropogenic activities [5]. Human exposure to harmful heavy metals can occur in many ways, ranging from the consumption of contaminated food, exposure to air-borne particles, and contact or consumption of contaminated water and accumulate over a period of time [3,4]. Water related diseases can often be attributed to exposure to elevated heavy metal concentrations of both organic and inorganic contaminants. Many of these compounds exist naturally, but their concentration has increased as a result of anthropogenic activities [6].

Although small-scale mining has contributed enormously to the economy of Ghana over the years, it has also negatively impacted the environment and human health [7]. The major environmental impact associated with mining is the persistent release of harmful and toxic substances such as mercury (Hg), lead (Pb), cadmium (Cd), arsenic (As), among others [7,8,9]. Various studies link the pollution of some surface and groundwater bodies in Ghana to gold mining activities [10,11,12,13]. Recently, gold mining in Ghana has become unpopular due to its significant contribution of Hg, Pb, and other heavy metals from activities such as mineral exploitation, ore transportation, smelting, refining, disposal of tailings, and wastewaters [12,13,14,15,16].

In artisanal and small-scale mining, it is common practice for waste material (tailings) to be removed and piled in large mounds at the mining site. These piles of tailings often contain heavy metals found in the ore and in many instances, also contain mercury waste that was used during the amalgamation of gold [17]. In such instances, these tailings are exposed to the elements and can be easily weathered, releasing toxic metals into the soil, adjacent water bodies and, ultimately, groundwater.

There is increasing evidence linking toxicants such as Hg, Pb, As, and Cd to the incidence of cognitive impairments, especially in children, and cancers of all sorts [18,19]. Heavy metal contamination is associated with deficiencies of some essential nutrients in the human body [20]. This, ultimately, can result in decreased immunological defenses, disabilities associated with malnutrition, intrauterine growth retardation, impaired psychosocial faculties, and high prevalence of upper gastrointestinal cancer rates [20]. High concentrations of lead, arsenic, and other heavy metals can affect the nervous system and kidneys, and may cause reproductive disorders, skin lesions, endocrinal damage, and vascular diseases [21].

In Northern Ghana, most rural communities depend largely on natural water bodies as sources of drinking water. Given that mining activities rely heavily on water resources and are associated with pollution of these same water bodies; several studies have been conducted in the southern part of Ghana to assess the effect of mining on sources of drinking water [22,23,24,25]. However, there is a paucity of information on the impact of artisanal gold mining on sources of drinking water in the northern parts of Ghana. The main thrust of this study was to (1) investigate the levels of heavy metals such as As, Hg, Cd, Zn, and Pb in Tinga and Nangodi mining communities, and (2) undertake a comparative assessment of the levels of the aforementioned toxic chemicals whose health effect has rarely been investigated.

## 2. Materials and Methods

### 2.1. Study Areas

**Figure 1:**
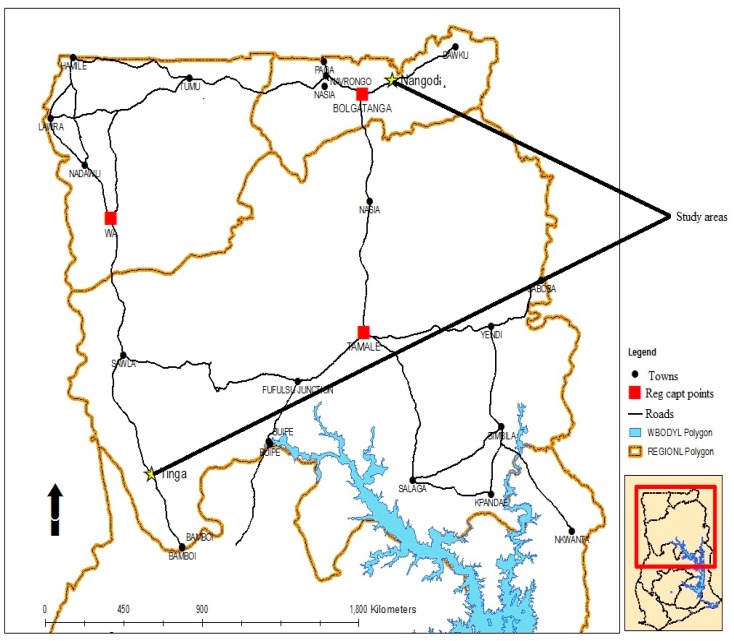
Map of northern Ghana showing Tinga and Nangodi communities.

The study was conducted in two mining communities located in two regions of northern Ghana. Nangodi is located in the Nabdam District of the Upper East Region (UER), in the eastern belt of the Nabdam Traditional area. The district, which located in the Guinean Savannah zone, lies between latitude 10°15′ and 10°60′ north of the equator and longitude 0°31′ and 1°05′ west of the Greenwich meridian (Figure 1). Tinga is located in the Bole–Bamboi District in the Northern Region, and also lies within the Guinea Savannah agro-ecological zone. It is located at N 8°35′59′′ and W −2°13′0′′. Tinga is located at the extreme western part of the Northern Region of Ghana (Figure 1). The geology in the vicinity of Tinga is primarily composed of granite and metamorphic rocks [25]. The Tinga and Nangodi communities (Bole-Nangodi belt) are remarkably similar in all aspects to rocks that occur in the northeast-trending belts in the country. Rocks are mainly dominated by birimian metavolcanics and metasedimentary rocks that are intruded by numerous granitoids, as well as felsic and mafic intrusives [26]. Metamorphism in the study areas varies from moderate greenschist to high-grade amphibolite facies [26]. The catchment area especially around Tinga, Kuri, Bombiri, Dakrupe, and Seripe has shown numerous prospects and occurrences of gold. Gold mineralization is mainly associated with shear zones, which cut across metavolcanics, metasedimentary, and granitic rocks [26].

### 2.2. Water Sampling

In both communities, water was sampled on a monthly basis for a period of six months (November 2011 to April 2012). In Nangodi, thirty (30) water samples were collected from boreholes, hand dug-out wells, and dug-outs in operation during the study within the catchment. A total of five samples were collected every month from the borehole, hand dug wells, and dug-outs; one sample from the borehole (BH) and two from the hand dug wells (HDWA and HDWB) and two from the dugouts (DA and DB) (Table 1).

At Tinga, water samples were collected from the only stream in the community (used for drinking and other domestic chores), which is also used for the washing of gold-bearing ore. Four boreholes which serve as a source of drinking water for the community were also selected for sampling. A total of seven samples were collected every month from the surface and groundwater sources; three samples from the stream (BGWP- before gold washing pool, GWP- gold washing pool, and AGWP- after gold washing pool) and four from boreholes (labelled BHT1, BHT2, BHT3, and BHT4) (Table 1). A total of 42 samples were collected from Tinga for analysis during the study period.

**Table 1:** Describing the sampling sites, sampling size and containers used for the sampling.

Sampling Site	Meaning of Label	No. of Samples
**Nangodi**		30
BHN	Borehole in Nangodi	6
HDWA	Hand dug-out well labelled A	6
HDWB	Hand dug-out well labelled B	6
DA	Dug-out labelled A	6
DB	Dug-out labelled B	6
**Tinga**		42
BHT1	Borehole at Tinga labelled 1	6
BHT2	Borehole at Tinga labelled 2	6
BHT3	Borehole at Tinga labelled 3	6
BHT4	Borehole at Tinga labelled 4	6
BGWP	before Gold Washing Pool in the Stream	6
GWPS	Gold Washing Pool in the Stream	6
AGWP	After Gold Washing Pool in the Stream	6

A total of 72 water samples were collected from twelve drinking water sources from the two mining communities within the six-month period. Sample bottles were rinsed with deionized water twice before samples were collected. One liter each of the samples collected were preserved with 0.5 mL of concentrated nitric acid (obtained from Water Research Institute laboratory, Tamale) and kept in ice-chests with ice packs at a temperature of 4 °C before transporting them to the Water Research Institute Laboratory in Tamale for analysis. The samples were then stored in a refrigerator at 4 °C until the analyses were completed.

### 2.3. Digestion of Samples for the Analyses of Hg, As, Pb, Zn and Cd

In the laboratory, the samples were filtered through Whatman 0.45 μm membrane filter paper. One hundred milliliters of the filtered water was mixed with 5 mL concentrated nitric acid (HNO_3_) and 5 mL concentrated sulphuric acid (H_2_SO_4_) [27]. To allow the acids to become concentrated, the mixture was heated until the volume was reduced to about 15 to 20 mL [24]. The digested sample was allowed to cool to room temperature. It was then filtered through Whatman’s 0.45 μm filter paper. The final volume was adjusted to 100 mL with double distilled water and stored for analysis [27].

### 2.4. Quality Assurance and Analysis

Strict quality assurance and quality control measures were adopted to ensure reliability of the results. All reagents and chemicals used were of high purity. Glasswares used during laboratory analysis were thoroughly cleaned with detergent and rinsed several times with deionized water. Dilutions were done using deionized water. A blank solution was read twenty-five times for the purposes of detection and quantification limits of the atomic absorption spectrophotometer (AAS), and the standard deviations were calculated for the noise levels generated for each of the elements of interest. The limit of detection (LOD) for each element was achieved as follows:

(1) $$LOD=\frac{3\times S}{m}$$

where **S** is the standard deviation of the blank readings and **m** represents the gradient of the calibration curve for each element. The limit of quantification was calculated using 10 × s/m. The accuracy and reproducibility of the analytical procedure was determined by spiking and homogenizing three replicates of each of three samples selected at random. Triplicate of each sample was spiked with three different concentrations of the element of interest as follows: Cd (0.5, 2.0 and 3.0 mg/L), Zn (0.25, 0.5 and 1.0 mg/L), and Pb (2.0, 5.0 and 10.0 mg/L) and treated in a similar manner as the samples. The absorbances measured by the AAS were converted to concentrations using standard calibration curves. One thousand milligrams per liter single element standards of the elements of interest, obtained from Fluka Analytical (Sigma-Aldrich Chemie GmbH, Buchs, Switzerland), were diluted using 10% HNO_3_ and used to generate the calibration curves for the AAS analysis. Trace metal (Hg, As, Pb, Zn, and Cd) analysis was done using the Shimadzu model AA 6300 in accordance with APHA [28].

Recovery and reproducibility studies, which are meant to check the sensitivity and efficiency of methods used in the chemical analysis, were conducted using certified standard reference solutions for Hg, Cd, Zn, As, and Pb manufactured by BDH Chemicals, UK. The percentage of Hg, Cd, Zn, As, and Pb recovered in the recovery studies were 98%, 93%, 94%, 100%, and 95%, respectively. Similar results were recorded for the reproducibility studies.

### 2.5. Principal Component Analysis

Principal component analysis (PCA) was used to compare the variability of heavy metals at the two study areas. Briefly, the PCA model is a linear grouping of variables explaining the variance structure of a matrix that condense the various data into a few principal components (PC). Considering a random vector X, which has a covariance matrix K with eigen values λ_1_ ≥λ_2_ ≥ … ≥ λ_n_ ≥ 0, and eigen vectors a_1_, a_2_, … a_n_. Considering a matrix X (n × p) a set of n observations on a vector of *p* variable:

(2) $$\{X_{1}X_{2}...X_{2}\}\in R^{p}$$

The linear combinations of the X matrix is constructed as follows:

(3) $$Z_{1}=a_{1}^{t}=a_{11}x_{1}+a_{12}x_{2}+...a_{p1}x_{n}$$

(4) $$Z_{2}=a_{2}^{t}=a_{21}x_{1}+a_{22}x_{2}+...a_{p2}x_{n}$$

(5) $$Z_{p}=a_{p}^{t}=a_{p1}x_{1}+a_{p2}x_{2}+...a_{pn}x_{n}$$

(6) $$Var[z_{i}]=a_{i}^{t}Kai,i=1,2,...n$$

(7) $$Cov[Zi,Zj]=a_{i}^{t}Kaj,i=1,2,...n,j=1,2,...n$$

The principal components are those uncorrelated linear combinations of Z_1_, Z_2_, … Z_i_ and each component is a linear weight combination which maximized variances as large as possible and ranked in descending order.

## 3. Results and Discussion

The study assessed the concentrations of mercury (Hg), arsenic (As), lead (Pb), zinc (Zn), and cadmium (Cd) obtained from surface and ground water bodies used for drinking and other domestic chores in Nangodi and Tinga in northern Ghana. Concentrations of the heavy metals studied are presented in Table 2 and Table 3.

**Table 2:** Mean concentration (mg/L) of heavy metals in water samples from Nangodi and Tinga.

Study Area	Metal	*n*	Minimum	Maximum	Mean	SD	WHO Limit ^a^
Nangodi (5 sites)	Mercury	30	0.001	0.191	0.038	0.001	0.010
	Arsenic	30	0.001	0.115	0.031	0.005	0.010
	Lead	30	0.005	0.791	0.250	0.008	0.010
	Zinc	30	0.005	786.0	0.034	0.001	3.000
	Cadmium	30	0.001	2.227	0.534	88.0	0.003
Tinga (7 sites)	Mercury	42	0.001	0.259	0.064	0.007	0.010
	Arsenic	42	0.001	0.003	0.002	0.001	0.010
	Lead	42	0.001	0.227	0.031	0.001	0.010
	Zinc	42	0.001	0.005	0.003	0.001	3.000
	Cadmium	42	0.002	0.071	0.023	0.008	0.003

Samples were collected from five and seven sample sites in Nangodi and Tinga, respectively (over six months), these concentrations are the general values and averages. ***n*** represents sample size; WHO limit **^a^** represents WHO 2008 permissible limit for drinking water.

### 3.1. Mercury

Mercury concentrations in water samples from Nangodi ranged from 0.001 to 0.191 mg/L with a mean of 0.038 ± 0.001 mg/L, whilst levels recorded in Tinga ranged from 0.010 to 0.259 mg/L with a mean concentration of 0.064 ± 0.007 mg/L (Table 2). Mean Hg levels in drinking water sources in both study areas were above World Health Organization (WHO) [29] permissible limit of 0.010 mg/L for drinking water (Table 2). Earlier studies conducted in the Nangodi catchment reported Hg concentrations that ranged from below detection limits to 0.190 mg/L [30] and also in Datuku, Hg levels ranging from 0.0002 mg/L to 0.020 mg/L were recorded [31]. Another study in Tinga reported Hg concentrations ranging from 0.010 to 0.230 mg/L [32]. A similar study conducted in Kibi traditional area, Ghana, where there is a current upsurge of artisanal mining activities, reported mean Hg concentrations of 0.010 mg/L (Obronikrom), 0.008 mg/L (Kibi-Deaf), 0.003 mg/L (Bunso) and 0.002 mg/L (Apapam) [33] that are lower that mean values of this present study.

Drinking water sources in Tinga recorded higher concentrations of Hg compared to that from Nangodi. This could be due to the washing of gold-bearing ores close to the water bodies. In Tinga, ores are transported from mining sites, crushed, washed, and the gold extracted with Hg very close to available water bodies. In Nangodi, the highest concentration of Hg was found in samples from HDWA (hand dug-out well labeled A) and DA (dug-out labeled A) where the gold amalgam is roasted (Table 3). The water bodies were polluted perhaps due to the direct washing of gold bearing ores in the area and the percolation of Hg-ladened waste water released from the washing bay. Generally, mercury concentrations were high in water bodies that were close to the mining activities in each community.

**Table 3:** Concentration (mg/L) of heavy metals in water samples from Nangodi and Tinga.

Sampling Sites		Hg	As	Pb	Zn	Cd
Nangodi (5 sites)						
BHN	Range	0.001–0.001	0.001–0.115	0.146–0.791	0.005–0.786	0.003–0.900
	Mean ± SD	0.005 ± 0.001	0.044 ± 0.008	0.440 ± 0.402	0.143 ± 0.008	0.335 ± 0.015
HDWA	Range	0.006–0.180	0.002–0.034	0.005–0.205	nd	0.034–0.786
	Mean ± SD	0.063 ± 0.006	0.010 ± 0.001	0.080 ± 0.010	nd	0.223 ± 0.310
HDWB	Range	0.014–0.070	0.007–0.022	0.020–0.769	0.005–0.018	0.001–1.600
	Mean ± SD	0.032 ± 0.005	0.014 ± 0.004	0.251 ± 0.050	0.009 ± 0.001	0.444 ± 0.970
DA	Range	0.012–0.191	0.047–0.109	0.034–0.618	0.005–0.020	0.121–0.910
	Mean ± SD	0.072 ± 0.008	0.065 ± 0.004	0.222 ± 0.096	0.008 ± 0.001	0.533 ± 0.020
DB	Range	0.005–0.051	0.002–0.091	0.005–0.508	nd	0.080–2.227
	Mean ± SD	0.020 ± 0.057	0.023 ± 0.035	0.223 ± 0.212	nd	1.090 ± 0.033
Tinga (7 sites)						
BHT1	Range	0.057–0.210	0.001–0.003	0.001–0.010	0.002–0.004	0.010–0.070
	Mean ± SD	0.127 ± 0.090	0.002 ± 0.001	0.008 ± 0.004	0.003 ± 0.001	0.040 ± 0.001
BHT2	Range	0.009–0.220	0.001–0.003	0.001–0.020	0.001–0.003	0.002–0.070
	Mean ± SD	0.070 ± 0.010	0.002 ± 0.001	0.010 ± 0.001	0.002 ± 0.001	0.040 ± 0.003
BHT3	Range	0.056–0.270	0.010–0.002	0.001–0.010	0.001–0.003	0.002–0.060
	Mean ± SD	0.127 ± 0.020	0.001 ± 0.001	0.008 ± 0.004	0.002 ± 0.001	0.027 ± 0.010
BHT4	Range	0.001–0.090	0.001–0.003	0.001–0.020	0.001–0.003	0.002–0.060
	Mean ± SD	0.040 ± 0.010	0.002 ± 0.001	0.010 ± 0.002	0.002 ± 0.001	0.034 ± 0.020
BGWP	Range	0.003–0.060	0.001–0.003	0.009–0.060	0.001–0.003	0.002–0.002
	Mean ± SD	0.027 ± 0.020	0.001 ± 0.001	0.035 ± 0.010	0.002 ± 0.001	0.011 ± 0.009
GWP	Range	0.020–0.040	0.001–0.002	0.024–0.227	0.003–0.005	0.002–0.010
	Mean ± SD	0.027 ± 0.010	0.001 ± 0.001	0.070 ± 0.027	0.004 ± 0.001	0.004 ± 0.002
AGWP	Range	0.001–0.060	0.001–0.007	0.024–0.227	0.002–0.005	0.002–0.010
	Mean ± SD	0.027 ± 0.002	0.003 ± 0.001	0.070 ± 0.001	0.004 ± 0.001	0.004 ± 0.001
WHO Limit ^a^		0.010	0.010	0.010	3.000	0.003

A total of 72 samples were collected from five and seven sample sites in Nangodi and Tinga, respectively (over six months), these concentrations is summary results of the various samples analyzed. BH represents borehole; HDWA, HDWB, DA and DB represents various samples collected from hand dug-outs wells and dug-outs; BGWP (before gold washing pool), GWP (gold washing pool) and AGWP (after gold washing pool) represent the various sampling points in the stream; BH1, BH2, BH3 and BH4 represent the various four boreholes where samples were collected; WHO limit ^a^ represents the WHO 2008 permissible limit for drinking water; nd—not detected.

### 3.2. Arsenic

Arsenic concentrations in the water samples from Nangodi ranged from 0.001 to 0.115 mg/L with a mean of 0.031 ± 0.005 mg/L (Table 2). Samples from Tinga ranged from 0.001 to 0.003 mg/L with a mean concentration of 0.002 ± 0.001 mg/L (Table 2) for arsenic, which were within the WHO permissible limit of 0.010 mg/L for drinking water [29]. Earlier, studies conducted in the Nangodi catchment reported As concentrations that ranged from below detection limits to 0.120 mg/L [30] and in Datuku As concentrations ranging from 0.002 mg/L to 0.004 mg/L were reported [31]. Asamoah-Boateng [33] reported As concentration that ranged from 0.010 to 0.090 mg/L from surface water samples in Newmont Ghana gold mining concession areas.

The maximum concentration of As found in sampling sites from Nangodi were in HDWA (hand dug-out well labelled A) and DA (dug-out labelled A), that exceeded the stipulated standard for drinking water, however, the least was recorded in a borehole (BHN) and a hand dug-out well (HDWB) (Table 3). Generally, all sampling sites (5) from Nangodi recorded mean As concentrations that exceeded the WHO stipulated limit [29], but some mean values recorded from Tinga samples (seven sites) did not exceed the standard for drinking water (Table 3). The high concentration could be due to the mining activities or runoff from agricultural areas, where materials containing arsenic such as fertilizer and pesticides were used. However, borehole and hand dug-out well labelled B in Nangodi, which were far from the mining processing recorded low levels of As. Water samples from Nangodi recorded higher levels of As compared to those from Tinga, probably because the water sources are located where active mining was taking place. In Tinga, however, mined rocks are conveyed from the mining center close to the stream where washing is done.

Arsenic in mined rocks and ore stockpiles heaped close to surface water bodies could be leached into the water bodies [34]. Arsenic is carcinogenic in all of its oxidation states and high-level exposure can cause death [35]. It is also mutagenic and teratogenic, if used in high environmental doses [36]. Chronic exposure to high levels of As in drinking water to humans and animals may lead to various health effects such as skin, internal cancers, cardiovascular, and neurological disorders [37,38].

### 3.3. Lead

The lead concentrations in samples from Nangodi ranged from 0.005 to 0.791 mg/L with a mean of 0.250 ± 0.008 mg/L (Table 2), however, samples from Tinga recorded lead concentrations ranging from 0.001 to 0.227 mg/L with a mean of 0.031 ± 0.001 mg/L (Table 2). Samples from Nangodi that recorded the lowest lead concentration were HDWA and DB whilst the highest concentration was recorded from sample DA (Table 3). Asamoah-Boateng [33] reported lead concentrations that ranged from 0 to 2.710 mg/L from surface waters samples in Newmont Ghana gold mining concession areas. Earlier study in Tinga reported Pb concentrations that ranged from below detection limits to 0.188 mg/L [32].

Almost all samples from Nangodi exceeded the WHO [29] stipulated limit of 0.010 mg/L for drinking water. However, Tinga samples recorded high values above the WHO permissible limit at various sampling points in the stream (Table 3). The maximum concentration of Pb in Tinga was recorded in the stream where the processing of the mined minerals was done (GWP: gold washing pool in the stream, and AGWP: after gold washing pool in the stream) and the minimum was recorded in the second borehole (BHT2). This suggests that runoff from domestic, industrial (such as improper disposal of acid lead batteries and wind-blown dust), and agricultural waste might be the main source of Pb pollution in the Tinga community. The high content of Pb in the stream could be due to weathering and leaching of lead from waste rocks dumps. Lead is one of the most significant toxicants of the heavy metals and the inorganic forms are absorbed through ingestion by food and water, and inhalation [39].

### 3.4. Zinc

The zinc concentrations recorded in samples from Nangodi ranged from 0.005 to 0.786 mg/L with a mean of 0.034 ± 0.001 mg/L (Table 2). Samples from Tinga recorded zinc values that ranged from 0.001 to 0.005 mg/L with mean concentration of 0.003 ± 0.001 mg/L (Table 2). The zinc concentrations recorded from both mining communities were within the WHO [29] permissible limit of 3.0 mg/L for drinking water. Similar study conducted in Datuku in Nangodi catchment reported zinc concentrations that ranged from below detection limits to 0.013 mg/L [31]. Asamoah-Boateng [33] reported zinc concentration that ranged from 0 to 0.190 mg/L from surface waters samples in Newmont Ghana gold mining concession areas. However, zinc concentrations were higher in samples collected from Nangodi as compared to Tinga samples (Table 3). Zinc is an important trace element that plays a vital role in the physiological and metabolic process of many organisms. Nevertheless, higher concentrations of zinc can be toxic to organisms [40].

### 3.5. Cadmium

The cadmium concentrations obtained from Nangodi samples ranged from 0.001 to 2.227 mg/L with a mean of 0.534 ± 0.088 mg/L (Table 2). Samples from Tinga recorded cadmium concentrations that ranged from 0.002 to 0.071 mg/L with a mean concentration of 0.023 ± 0.008 mg/L (Table 2). The mean Cd concentrations of all sampling sites from both mining communities exceeded the WHO [29] permissible limit of 0.003 mg/L for drinking water. A similar study conducted in artisanal gold mining communities within the Kibi traditional area, Ghana, reported mean values of cadmium in river water at Apapam, Bunso, Kibi-Deaf, and Obronikrom as 0.006, 0.008, 0.008, and 0.010 mg/L, respectively [33]. Earlier study in Nangodi catchment reported Cd concentrations that ranged from below detection limit to 0.223 mg/L [30] and in Datuku, Cd concentrations ranged from below detection limits to 1.700 mg/L [31]. Additionally, an earlier study in Tinga reported Cd concentration that ranged from 0.002 to 0.103 mg/L [32]. In Nangodi, the lowest concentration was recorded from sampling site HDWB whilst the highest concentration was recorded from DB (dugout labeled B) (Table 3). Samples from Tinga recorded the highest value from BH2 (Table 3). However, it is clear from the mean values of samples from Nangodi recorded higher Cd levels compared to samples from the Tinga community. The residents of Nangodi and Tinga cannot be risk-free from cadmium. Cadmium causes adverse health effects such as kidney damage, bronchitis, and osteomalacia (soft bones) at very low exposure levels [41]. Cadmium affects the nervous system, causes damage to DNA and the immune system, and enhances the development of cancer. It can also cause other non-cancerous diseases that include loss of sense of smell and taste, fibrosis, upper respiratory diseases, shortness of breath, skeletal effects, lumbago, hypertension, tubular proteinuria, and cardiovascular diseases [28].

### 3.6. Principal Component Analysis (PCA)

In this study, principal component analysis (PCA) was performed to identify possible variabilities in heavy metals measured in sources of drinking water in the two communities. In all, five components with eigenvalues greater than 1 were extracted, accounting for 73.7% of total variance. The first three components accounted for more than 50.0% of total variance, with PC1 (Cdng, Pbtg, and Hgtg) accounting for 22.0% of total variance. PC2 (Znng and Asng) accounted for 15.5% of total variance, whilst PC3, which was made up of Hgng and Zntg, accounted for 13.5% of total variance.

**Figure 2:**
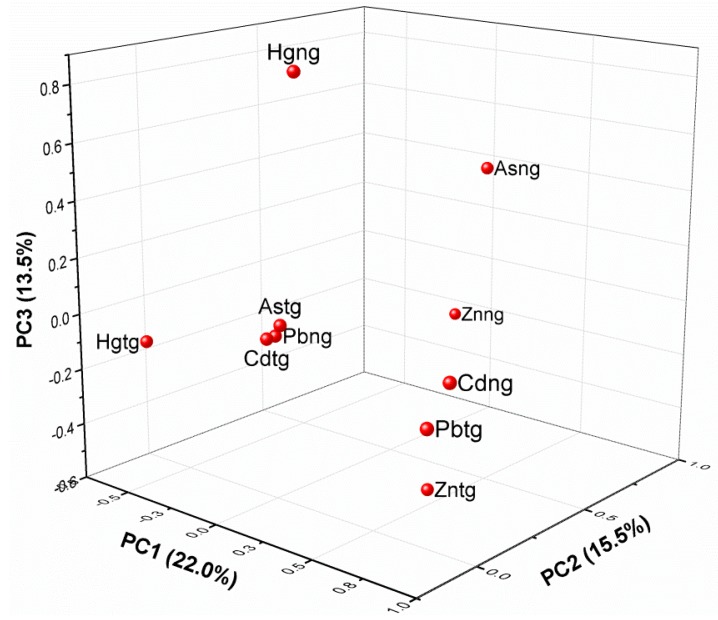
Principal component analysis (PCA) of heavy metals (Hg, Pb, As, Cd, and Zn) in the Nangodi (ng) and Tinga (tg) communities in northern Ghana. The first three components (PC1, PC2, and PC3) were more than 50.0% of total variance.

The first component (PC1), which generally explained the majority of total variance (22.0%), had high loadings on Cdng, Pbtg, and Hgtg. This represented the first distinction in the profiles of heavy metals in the two communities (Figure 2). It suggests that, mining activities in the study areas has contributed to the high loading of metals such as Cd (Nangodi), Hg (Tinga), and Pb (Tinga) which could come from anthropogenic activities such as mining [42]. Additionally, Hgng and Hgtg were observed to be well isolated from other heavy metals in the two communities, suggesting that they emanate from sources different from that of the other metals. The findings of this study compares favorably with that of [42] which suggests that they come from a different source compared to the other heavy metals. Hg is usually applied in the extraction process by small scale miners in the communities, whereas metals such as Cd, As, Zn, and Pb are mostly leached from excavated mineral-bearing ores. The rampant and indiscriminate use of Hg in gold extraction has been recorded in parts of the country [43]. Heavy metals like As (Tinga), Pb (Nangodi), Cd (Tinga), which are in one group and Zn (Nangodi), Cd (Nangodi), Pb (Tinga), and Zinc (Tinga), which were in the other, showed that these metals correlate and are influenced in a similar way. This may suggests that they emanate from the same source and have a close physiological relationship [44].

## 4. Conclusions

It is evident from the present study that direct drinking of water from these water sources can be deleterious to consumers as levels of some heavy metals were above the WHO stipulated limits. Generally, levels of Hg, As, Pb, Zn, and Cd in water from Nangodi exceeded WHO stipulated limits of 0.010 for Hg, As, and Pb, 3.0 for Zn and 0.003 for Cd, while that of Hg, Pb, and Cd were higher in drinking water sampled Tinga. Though there are probable sources that may account for the presence of heavy metals in the communities, activities of small scale miners seem to contribute to the elevation. Principal component analysis showed that metals like Hg in the two mining communities emanated from a different source (possibly from anthropogenic sources) compared to other metals like Zn, Pb, Cd, and As. Based on the findings of this study, it is recommended that more studies are conducted to assess the contributions of mining to heavy metals in these two communities.
